# Identification of iron metabolism-related genes as diagnostic signatures in sepsis by blood transcriptomic analysis

**DOI:** 10.1515/biol-2022-0549

**Published:** 2023-02-09

**Authors:** Huijun Li, Xu Wang, Qing Yang, Liming Cheng, Hao-Long Zeng

**Affiliations:** Department of Laboratory Medicine, Tongji Hospital, Tongji Medical College, Huazhong University of Science and Technology, Wuhan, China; Institute of Food Science and Engineering, Wuhan Polytechnic University, Wuhan, China

**Keywords:** sepsis, iron metabolism, diagnostic signatures, transcriptomic analysis, lipocalin 2

## Abstract

Iron metabolism is considered to play the principal role in sepsis, but the key iron metabolism-related genetic signatures are unclear. In this study, we analyzed and identified the genetic signatures related to the iron-metabolism in sepsis by using a bioinformatics analysis of four transcriptomic datasets from the GEO database. A total of 21 differentially expressed iron metabolism-related signatures were identified including 9 transporters, 8 enzymes, and 4 regulatory factors. Among them, lipocalin 2 was found to have the highest diagnostic value as its expression showed significant differences in all the comparisons including sepsis vs healthy controls, sepsis vs non-sepsis diseases, and mild forms vs severe forms of sepsis. Besides, the cytochrome P450 gene CYP1B1 also showed diagnostic values for sepsis from the non-sepsis diseases. The CYP4V2, LTF, and GCLM showed diagnostic values for distinguishing the severe forms from mild forms of sepsis. Our analysis identified 21 sepsis-associated iron metabolism-related genetic signatures, which may represent diagnostic and therapeutic biomarkers of sepsis, and will improve our understanding of the molecular mechanism underlying the occurrence of sepsis.

## 1 Introduction

Sepsis is defined as life-threatening organ dysfunctions because of the dysregulated host response to infection [1]. As the prevalence increases, sepsis affects tens of millions of people worldwide, leading to high mortality and morbidity [2]. Clinical management of patients with sepsis relies mainly on early recognition, so that correct therapeutic intervention including treatment of the infection and initial resuscitation procedures could be started rapidly [3,4]. In the past decades, the pathogenesis of sepsis and the associated factors as well as their regulation mechanisms have been increasingly understood; however, new and more specific biomarkers are still lacking, and non-critically ill and more effective treatments still remain to be developed [5].

Iron is a crucial trace element that is essential for fundamental processes in both humans and bacteria. In sepsis, iron metabolism is altered, including increased iron transport and uptake into cells and decreased iron export. This intracellular sequestration of iron limits its availability to circulating pathogens, which serves as a conservative strategy against the pathogens [6].

During iron metabolic biological process, iron participates in oxidation–reduction reactions, and catalyzes the reactive oxygen generation, therefore the increase in labile iron may cause oxidative injury and cell death, such as ferroptosis. Ferroptosis is a novel form of cell death characterized by the iron-dependent accumulation of lipid peroxides, the most prominent feature is plasma membrane damage caused by the generation of lipid peroxides [7]. Moreover, iron performs its functions as a constituent of proteins in the form of heme, iron-sulfur clusters, ferritin, or other functional groups, thereby targeting and limiting iron’s reactivity [6]. In sepsis, hemolysis results in the release of free hemoglobin (Hb) into the plasma, and Hb disassembles and auto-oxidizes, releasing heme and iron, which increases inflammation and cell death, exacerbating the damage to the body and increasing the risk of death [8]. The iron metabolism disorders are substantial and correlate with the severity of sepsis. This suggests that iron metabolism-related genes may be useful as a diagnostic signature for sepsis and for evaluating the disease severity.

In the present study, we identified the genetic signatures related to the iron metabolism in sepsis by using a bioinformatics analysis of four transcriptomic datasets downloaded from the GEO database. A total of 21 differentially expressed iron metabolism-related signatures were identified, including 9 transporters, 8 enzymes, and 4 regulatory factors, among which lipocalin 2 (LCN2) showed consistent differences in all the comparisons including sepsis vs healthy groups, sepsis vs non-sepsis groups, and mild vs severe forms of sepsis. Our results identified the sepsis associated iron metabolism-related genetic signatures, which may represent diagnostic and therapeutic biomarkers of sepsis, and the knowledge of these genes will improve our understanding of the molecular mechanism underlying the occurrence of sepsis.

## 2 Materials and methods

### 2.1 Iron metabolism-related gene sets

Iron metabolism-related genes were retrieved from 16 gene sets downloaded from the Molecular Signatures Database (MSigDB), including the GO_IRON_ION_BINDING, GO_2_IRON_2_SULFUR_CLUSTER_BINDING, GO_4_IRON_4_SULFUR_CLUSTER_BINDING, GO_IRON_ION_IMPORT, GO_IRON_ION_TRANSPORT, GO_IRON_COORDINATION_ENTITY_TRANSPORT, GO_RESPONSE_TO_IRON_ION, MODULE_540, GO_IRON_ION_HOMEOSTASIS, GO_CELLULAR_IRON_ION_HOMEOSTASIS, GO_HEME_BIOSYNTHETIC_PROCESS, HEME_BIOSYNTHETIC_PROCESS, GO_HEME_METABOLIC_PROCESS, HEME_METABOLIC_PROCESS, HALLMARK_HEME_METABOLISM, and REACTOME_IRON_UPTAKE_AND_TRANSPORT. A total of 514 genes were extracted from the iron metabolism-related gene sets after removing overlapping genes (Table S1).

### 2.2 Patient datasets

Patient datasets were selected according to the following criteria: age ≥18 years, presence of control group (healthy and/or uncomplicated infections), peripheral blood as samples for gene expression profiling, submission date within 5 years (2017–2022). Finally, four gene expression datasets were selected and downloaded from the GEO database (https://www.ncbi.nlm.nih.gov/geo/) (Table 1), including two expression profiling by array: GSE69063 and GSE134347 [9,10], and two expression profiling by high throughput sequencing: GSE154918 and GSE185263 [11,12]. In GSE69063 datasets, a total of 68 samples were collected from 30 patients with mild sepsis, 27 patients with severe sepsis, and 11 healthy controls. In GSE134347 datasets, 298 samples were collected from 256 patients with sepsis, 59 patients with noninfectious disease, and 83 healthy subjects. In GSE154918 datasets, 91 samples were collected from 20 patients with sepsis, 19 patients with sepsis shock, 12 patients with uncomplicated infections, and 40 healthy subjects. In GSE185263 datasets, 392 samples were collected from 345 patients with sepsis (293 survived, 52 dies) and 47 healthy subjects.

**Table 1:** Information of the patient datasets

Datasets	Cases, *N*	Samples	Experiment type	Reference
GSE69063	68	Whole blood	Array	[9]
Healthy	11			
Mild sepsis	30			
Severe sepsis	27			
GSE134347	298	Whole blood	Array	[10]
Healthy	83			
Noninfectious disease	59			
Sepsis	156			
GSE154918	91	Whole blood	High throughput sequencing	[11]
Heathy control	40			
Uncomplicated infection	12			
Sepsis	20			
Septic shock	19			
GSE185263	392	Whole blood	High throughput sequencing	[12]
Healthy	47			
Survived	293			
Died	52			

### 2.3 Differential analysis

Differentially expressed genes (DEGs) were calculated for each of the four datasets, between sepsis and control (healthy or noninfectious) samples using Limma package with a threshold of adjusted *p*-values of <0.05 and an absolute fold change >1.5 [13]. DEGs were also calculated between different subsets of sepsis (sepsis shock vs sepsis for GSE154198, severe sepsis vs mild sepsis for GSE69063). Next we identified the differentially expressed iron metabolism-related genes in sepsis by intersecting the MSigDB iron metabolism-related gene sets and the DEGs from the four datasets.

Hierarchical clustering was applied to explore the differences in expression patterns between samples of sepsis patients or healthy controls, and also between the differentially expressed iron metabolism-related genes, in the four datasets, respectively, using the heatmap package in R. The average linkage method was used to conduct the hierarchical clustering and Pearson correlation method was used to set the clustering distance.

### 2.4 Functional enrichment and protein–protein interaction (PPI) analysis

Functional enrichment analysis of differentially expressed iron metabolism-related genes was based on Gene Ontology (GO) database from molecular function, cellular component, and biological process using R package ClusterProfiler [14]. The PPI network was conducted via STRING database (https://string-db.org/). Cytoscape 3.7.2 (https://js.cytoscape.org/) was used to visualize PPI networks. The hub genes were screened using the Cytoscape software plugin cytoHubba. The gene–gene interaction network for hub genes was further constructed using Cytoscape software plugin GeneMANIA (http://www.genemania.org).

### 2.5 Statistical analysis

All statistical analyses in this study were conducted using R software (version 4.2.1). The receiver operating characteristic (ROC) analysis was performed and the area under curve (AUC) was calculated to evaluate the diagnostic performance of the genetic signatures. All the values of AUC with 95% confidence interval for the ROC analysis are listed in Tables S2 and S3. A two-tailed *p* value < 0.05 was considered statistically significant.

## 3 Results

### 3.1 Differentially expressed iron metabolism-related genes in patients with sepsis

To identify the sepsis-associated genes, we first analyzed the DEGs in whole blood samples between sepsis and non-sepsis, and also between the severe forms and mild forms of sepsis. In GSE69063, we totally identified 5,294 DEGs among severe sepsis, mild sepsis, and healthy controls, by using 68 samples collected from 30 patients with mild sepsis, 27 patients with severe sepsis, and 11 healthy controls (Figure S1). In GSE154198, 4,040 DEGs were identified among sepsis, sepsis shock, and healthy controls, by using 79 samples collected from 20 patients with sepsis, 19 patients with sepsis shock, and 40 healthy subjects (Figure S2). In GSE134347, 2,166 DEGs were identified between sepsis and noninfectious controls, or between sepsis and healthy controls, by using 298 samples collected from 256 patients with sepsis, 59 patients with noninfectious disease, and 83 healthy subjects (Figure S3). In GSE185263, we totally identified 7,567 DEGs between sepsis and healthy controls, by using 392 samples collected from 345 patients with sepsis and 47 healthy subjects (Figure S4).

**Figure 1:**
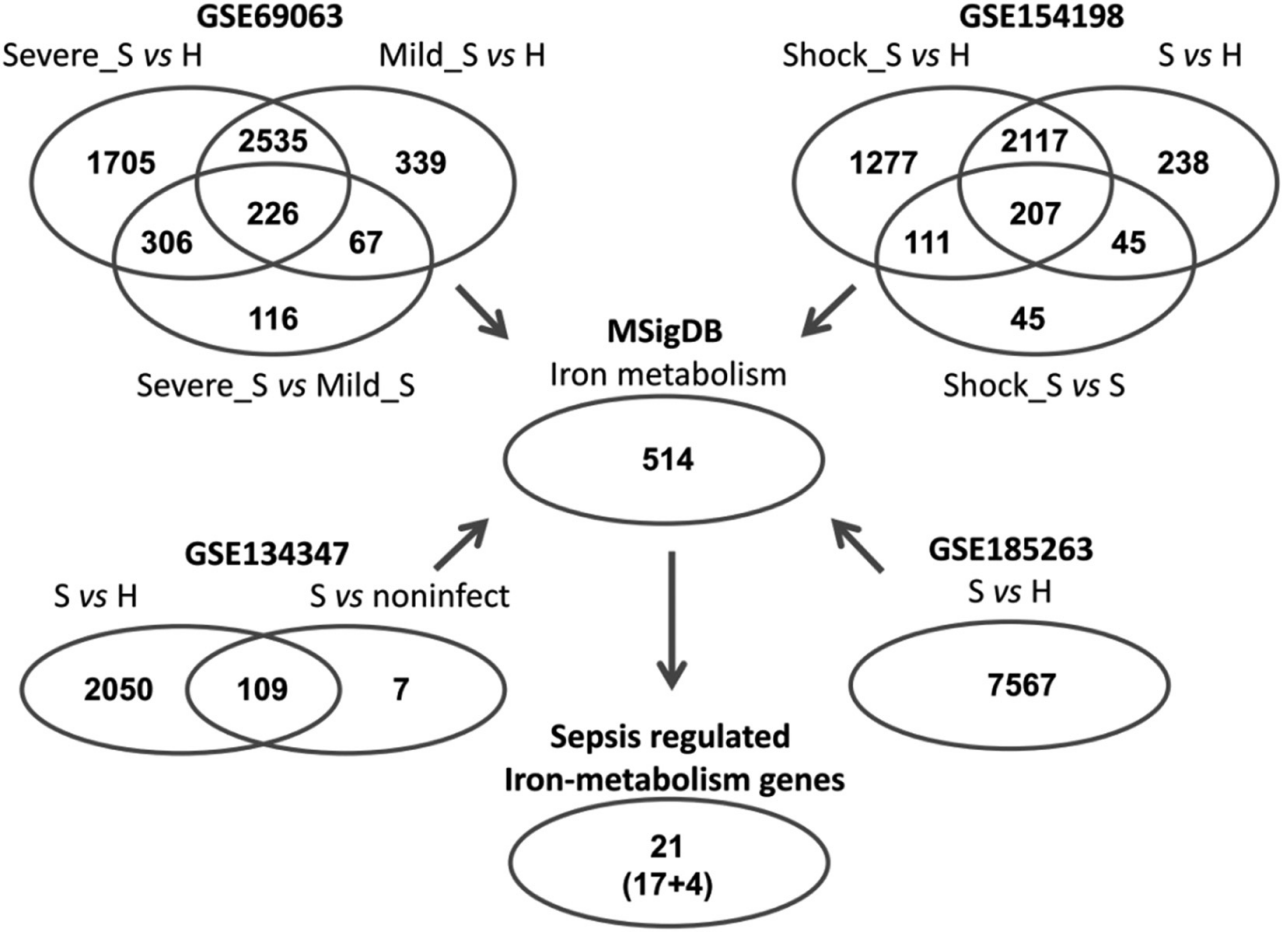
Identification of differentially expressed iron metabolism-related genes in sepsis by using the four transcriptomic datasets. A total of 5,294 DEGs among severe sepsis (Severe_S), mild sepsis (Mild_S), and healthy controls (H) were identified in GSE69063 dataset. A total of 4,040 DEGs among sepsis (S), sepsis shock (Shock_S), and healthy controls (H) were identified in GSE154198 dataset. A total of 2,166 DEGs between sepsis (S) and noninfectious controls (noninfect), or between sepsis (S) and healthy controls (H) were identified in GSE134347 dataset. A total of 7,567 DEGs between sepsis (S) and healthy controls (H) were identified in GSE185263 dataset. By extracting the iron metabolism-related genes, 21 genes were identified and differentially expressed simultaneously in all the four databases.

Next we extracted the iron metabolism-related genes from the DEGs in the four GEO databases, and identified a total of 21 sepsis related iron metabolism-related genes which were differentially expressed simultaneously in all the four databases (Figure 1). Among them, 17 genes were up-regulated including SLC22A4, MBOAT2, TSPO, NFE2, GCLM, FLVCR2, ATP6V1C1, CYP1B1, LCN2, AHSP, CLIC2, LTF, ELL2, CISD2, CA1, RHAG, and GYPA, while 4 genes were down-regulated including ALOX15, BCL2, FTO, and CYP4V2 in sepsis compared with the non-sepsis or healthy controls. These 21 genes included 9 transporters, 8 enzymes, and 4 regulatory factors. The detailed information of the 21 sepsis related iron metabolism-related genes are listed in Table 2.

**Table 2:** Changed iron-metabolism genes intersected from the four datasets

Gene	Changes in sepsis	Name	Function	Category
SLC22A4	↑	Solute carrier family 22, member 4	An organic cation transporter	Transporter
MBOAT2	↑	Membrane bound O-acyltransferase domain containing 2	An acyltransferase	Enzyme
TSPO	↑	Translocator protein	Binds protoporphyrin and helps transport porphyrins and heme	Transporter
NFE2	↑	Nuclear factor, erythroid 2	NF-E2 complex, participates in Hb production and heme synthesis	Regulatory factor
GCLM	↑	Glutamate-cysteine ligase modifier subunit	The first-rate limiting enzyme of GSH synthesis	Enzyme
FLVCR2	↑	Feline leukemia virus subgroup C receptor-related protein 2	An importer of heme	Transporter
ATP6V1C1	↑	ATPase H^+^ transporting V1 subunit C1	A V-ATPase subunit, a multisubunit enzyme	Enzyme
CYP1B1	↑	Cytochrome P450 family 1 subfamily B member 1	A cytochrome P450 monooxygenase	Enzyme
LCN2	↑	Lipocalin 2, or neutrophil gelatinase-associated lipocalin	An iron-trafficking protein	Transporter
AHSP	↑	Alpha-Hb-stabilizing protein	A chaperone to prevent the harmful aggregation of alpha-Hb, to modulate pathological states of alpha-Hb excess	Regulatory factor
CLIC2	↑	Chloride intracellular channel protein 2 ·	A component of chloride ion channels	Transporter
LTF	↑	Lactotransferrin	A major iron-binding and multifunctional protein	Transporter
ALOX15	↓	Arachidonate 15-lipoxygenase	A non-heme iron-containing dioxygenase	Enzyme
BCL2	↓	Apoptosis regulator Bcl-2	Acts as an inhibitor of autophagy and suppresses apoptosis by controlling the mitochondrial membrane permeability	Regulatory factor
FTO	↓	Alpha-ketoglutarate-dependent dioxygenase FTO	RNA demethylase that mediates oxidative demethylation of RNA species, and acts as a regulator of energy homeostasis	Enzyme
CYP4V2	↓	Cytochrome P450 family 4 subfamily V member 2	A cytochrome P450 monooxygenase	Enzyme
ELL2	↑	RNA polymerase II elongation factor ELL2	An elongation factor component of the SEC, involved in snRNA transcription	Regulatory factor
CISD2	↑	CDGSH iron-sulfur domain-containing protein 2	A zinc finger protein binds an iron/sulfur cluster and may be involved in calcium homeostasis	Transporter
CA1	↑	Carbonic anhydrase 1	Carbonic anhydrases	Enzyme
RHAG	↑	Rh associated glycoprotein	Associated with rhesus blood group antigen expression, participate in transmembrane transport of ammonium and carbon dioxide	Transporter
GYPA	↑	Glycophorin A	A major intrinsic membrane protein of the erythrocyte	Transporter

**Figure 2:**
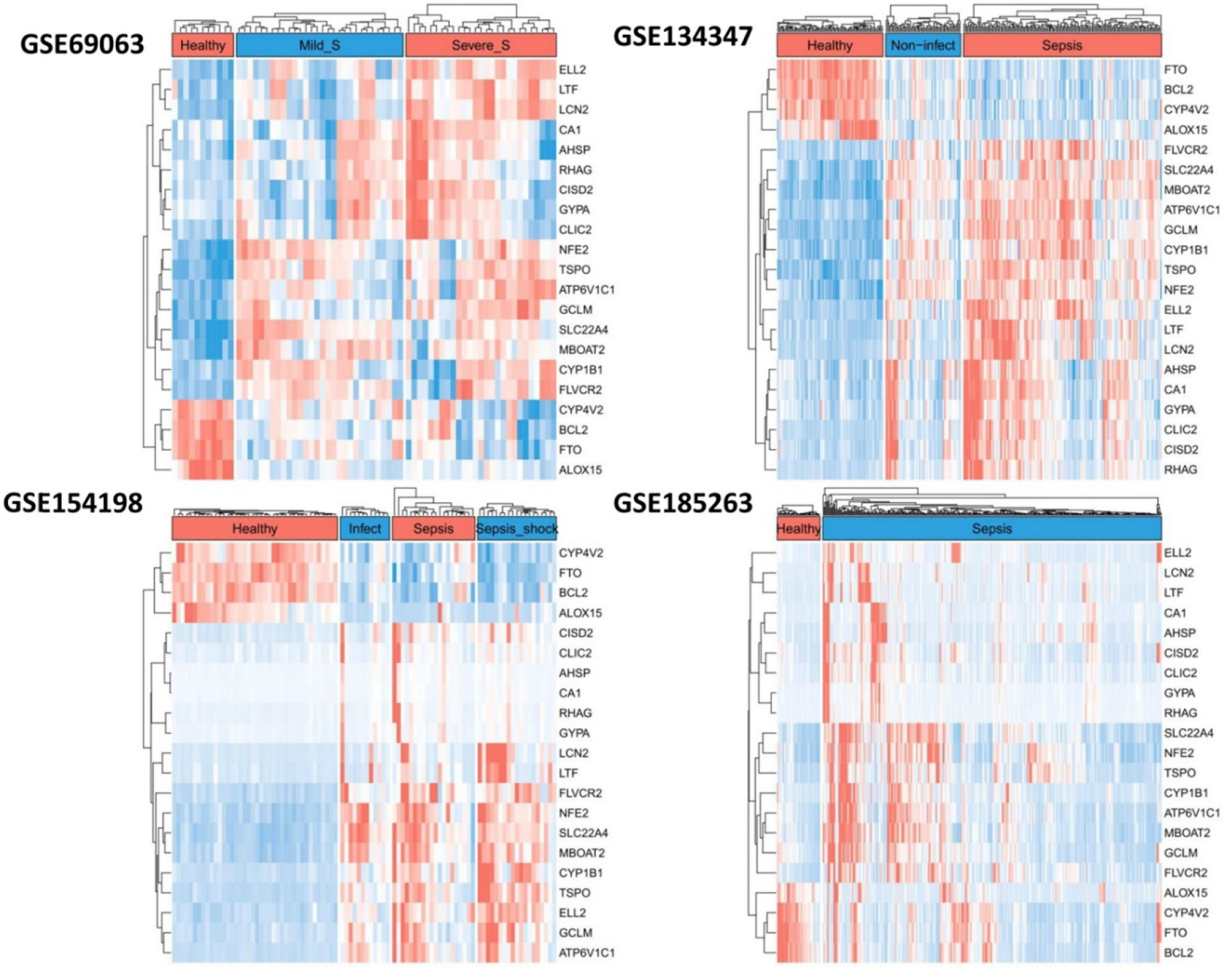
Heatmap of the expression levels of the sepsis related iron metabolism-related genes in the four transcriptomic datasets.

A fold-change represented color map was further built based on all the samples in the four GEO datasets, respectively, by using the 21 sepsis related iron metabolism-related genes (Figure 2). The expression patterns of 17 genes up-regulated and 4 genes down-regulated in sepsis compared to non-sepsis could be obviously found consistent in all the four color maps.

### 3.2 Functional enrichment and network analysis

**Figure 3:**
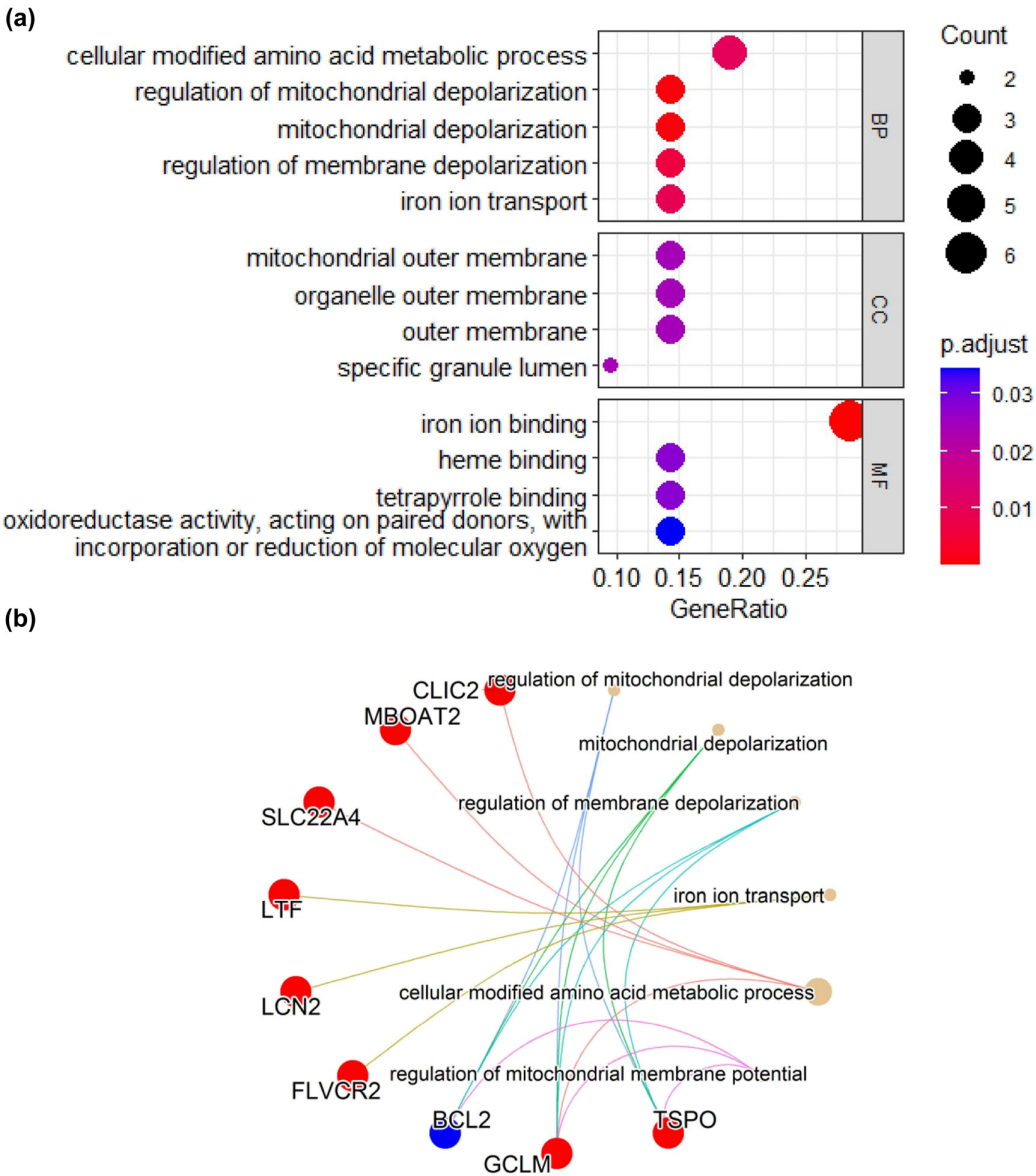
Enriched GO terms associated with the sepsis related iron metabolism-related genes. (a) GO results of biological process (BP), cellular component (CC), and molecular function (MF). (b) gene-concept network. Solid red circle represented up-regulated genes, while solid blue circle represented down-regulated genes.

Functional enrichment analyses were performed to explore the functions of the 21 DEGs. These genes were significantly enriched in biological process including cellular modified amino acid metabolic process (SLC22A4, MBOAT2, CLIC2, and GCLM), mitochondrial depolarization (BCL2, GCLM, and TSPO), and molecular functions of iron ion binding (LCN2, LTF, and FLVCR2), and mainly enriched in cellular component of mitochondrial or organelle membrane (Figure 3).

**Figure 4:**
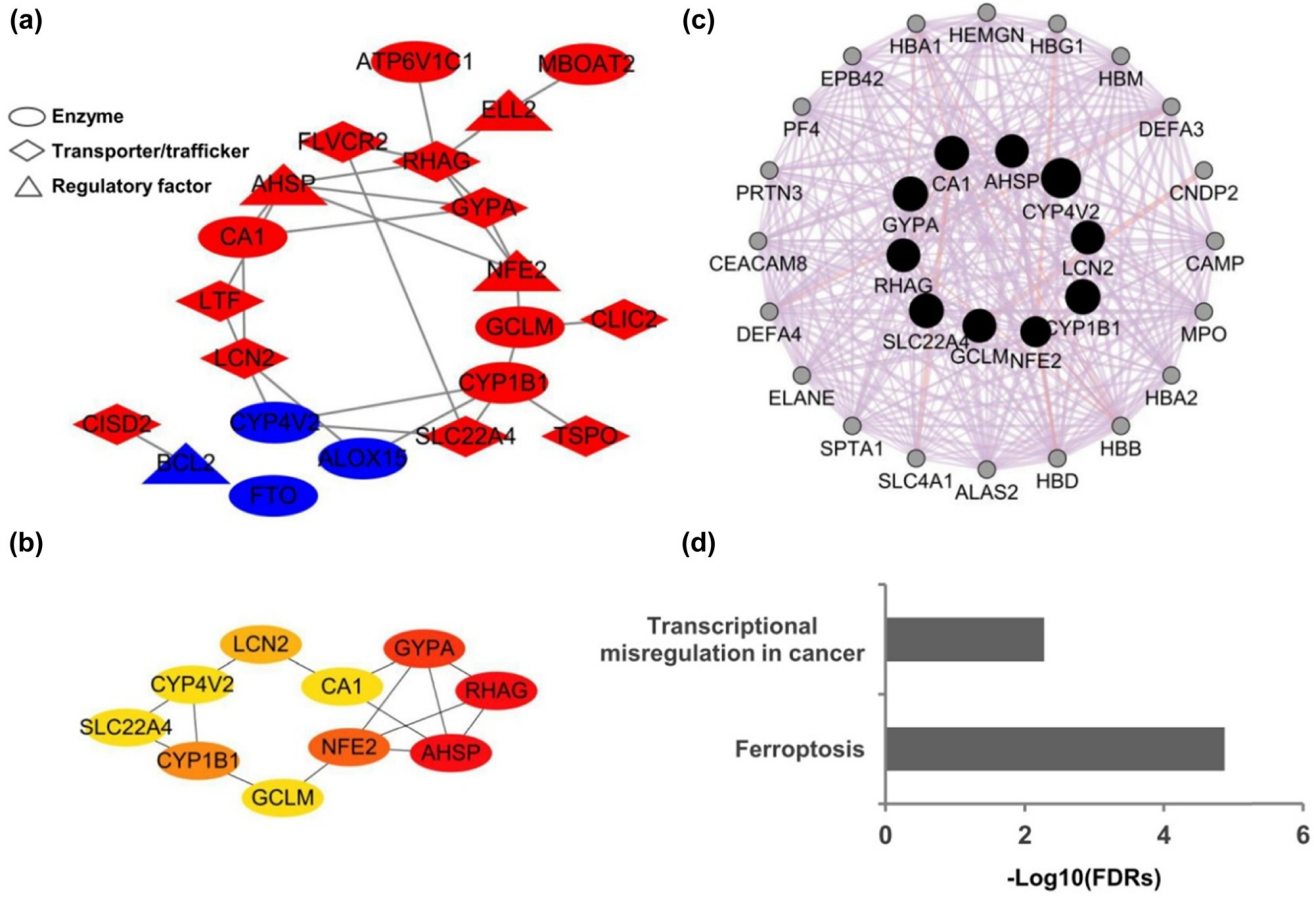
PPI network of the sepsis-related iron metabolism-related genes. (a) The 21 nodes and 27 edges in a PPI network were built among the 21 common DEGs including 9 transporters, 8 enzymes, and 4 regulatory factors. (b) The ten hub genes were further identified by using the cytoHubba. (c) Gene–gene interaction networks of the ten hub genes in GeneMANIA. Another 20 nodes representing genes and 265 links associated with the hub genes in co-expression and physical interactions were identified. (d) Enriched pathways of the ten hub genes and their interacted genes.

To further explore the potential interactions among these differentially expressed iron metabolism-related genes, a PPI network analysis was conducted with the STRING database. The 21 nodes and 27 edges in a PPI network were built among the 21 common DEGs (Figure 4a). Among these genes, the hub genes were further screened, which included SLC22A4, CYP4V2, CYP1B1, LCN2, GCLM, CA1, NFE2, GYPA, AHSP, and RHAG (Figure 4b). Then, we further applied GeneMANIA to identify the other potentially associated genes with these hub genes, and to construct their associated networks. We finally identified another 20 nodes representing genes and 265 links associated with the above 10 hub genes in co-expression and physical interactions (Figure 4c). These associated genes included TP53, GCLC, DEFA4, CAMP, EPB42, CEACAM8, MPO, ALAS2, CPs, SPTA1, ELANE, PF4, HBB, HBD, HBM, HEMGN, DEFA3, DDX6, SAE1, and ICAM2. Further pathway enrichment analysis revealed that these genes were significantly enriched in Ferroptosis (FDR = 1.34 × 10^–5^) (Figure 4d).

### 3.3 Genetic signatures distinguished sepsis from healthy and non-sepsis controls

**Figure 5:**
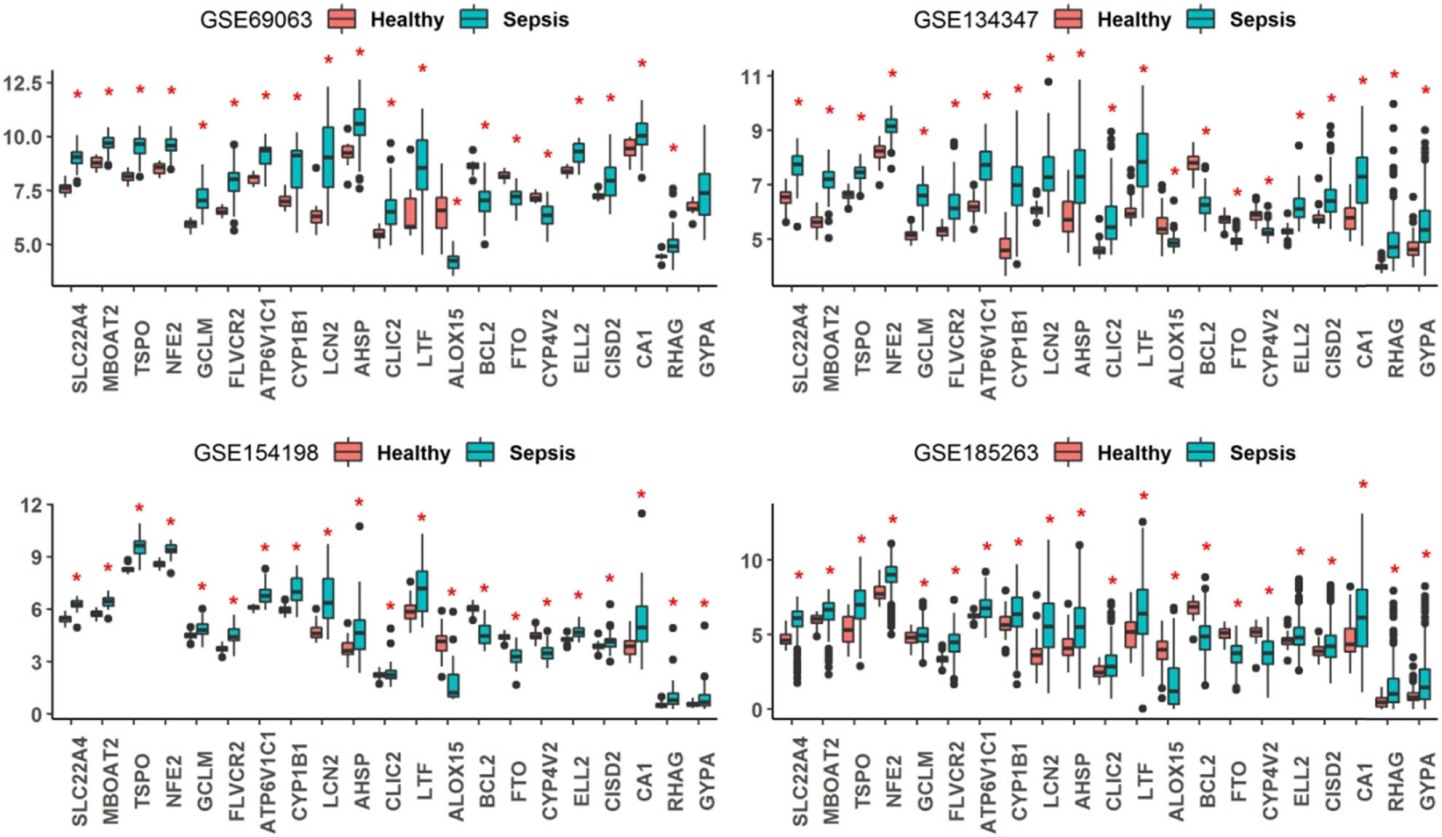
Expression levels of the sepsis related iron metabolism-related genes in patients with sepsis and healthy controls. *p < 0.05.

**Figure 6:**
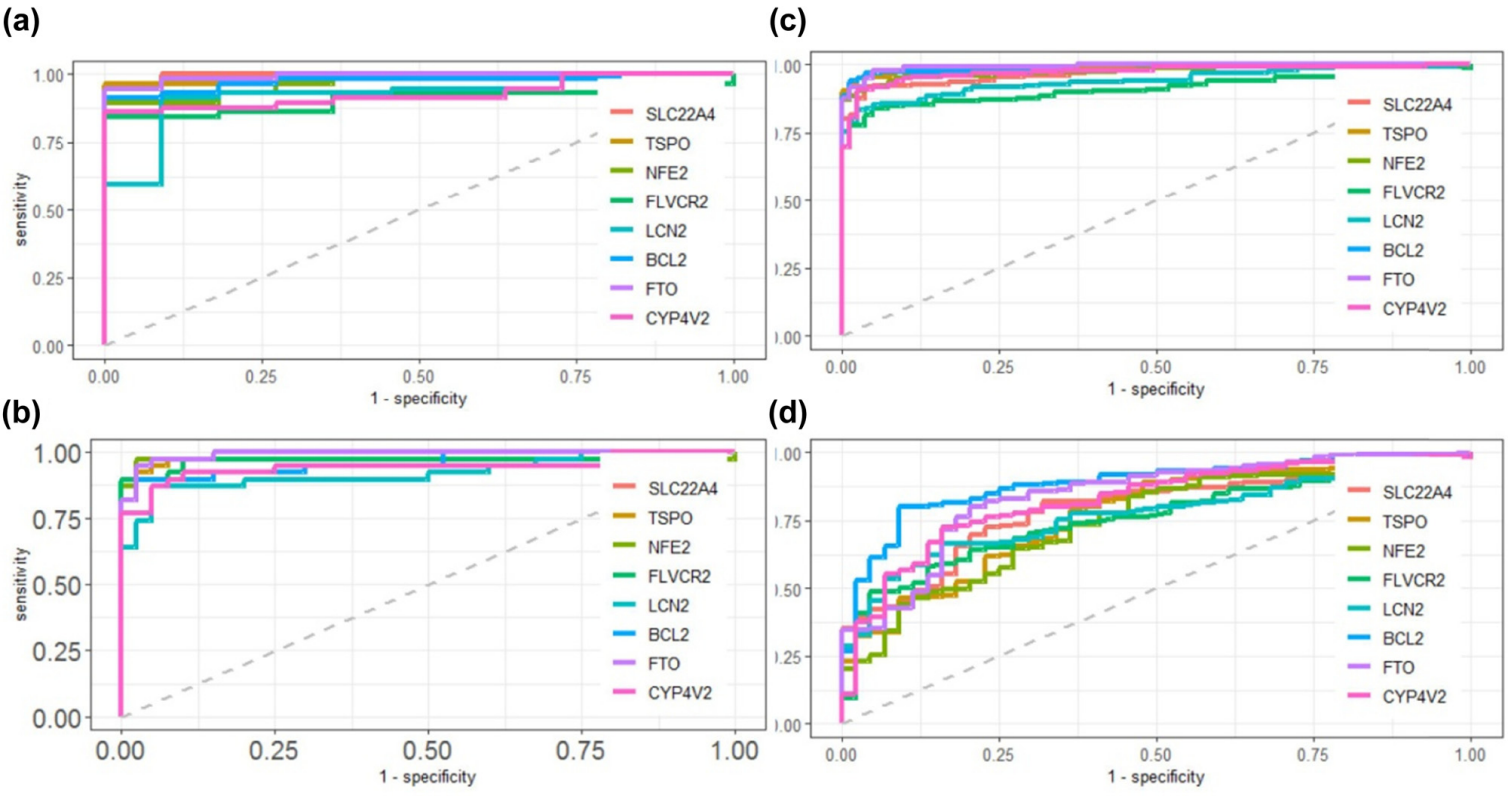
ROC curves of top eight iron metabolism-related genes including SLC22A4, TSPO, NFE2, FLVCR2, LCN2, BCL2, FTO, and CYP4V2 in the diagnosis of sepsis compared with healthy controls. The AUC was >0.9 in GSE69063 (a), GSE154918 (b), GSE134347 (c) datasets, and AUC > 0.75 in GSE69063 (d) dataset (Table S2).

To explore the diagnostic values of these genetic signatures in distinguishing sepsis from healthy controls, the expression levels of the 21 iron metabolism-related genes were compared between sepsis and healthy controls for the four GEO datasets, respectively (Figure 5). As expected, the 17 up-regulated genes and 4 down-regulated genes were consistently changed (*p* < 0.05) in all the four datasets. The performance of the ROC in terms of sepsis vs healthy controls was analyzed, and all the AUC are listed in Table S2. The ROC curve of the top eight iron metabolism-related genes including SLC22A4, TSPO, NFE2, FLVCR2, LCN2, BCL2, FTO, and CYP4V2 are shown in Figure 6, which had AUC > 0.9 in GSE69063, GSE134347, and GSE154198, and AUC > 0.75 in GSE69063.

**Figure 7:**
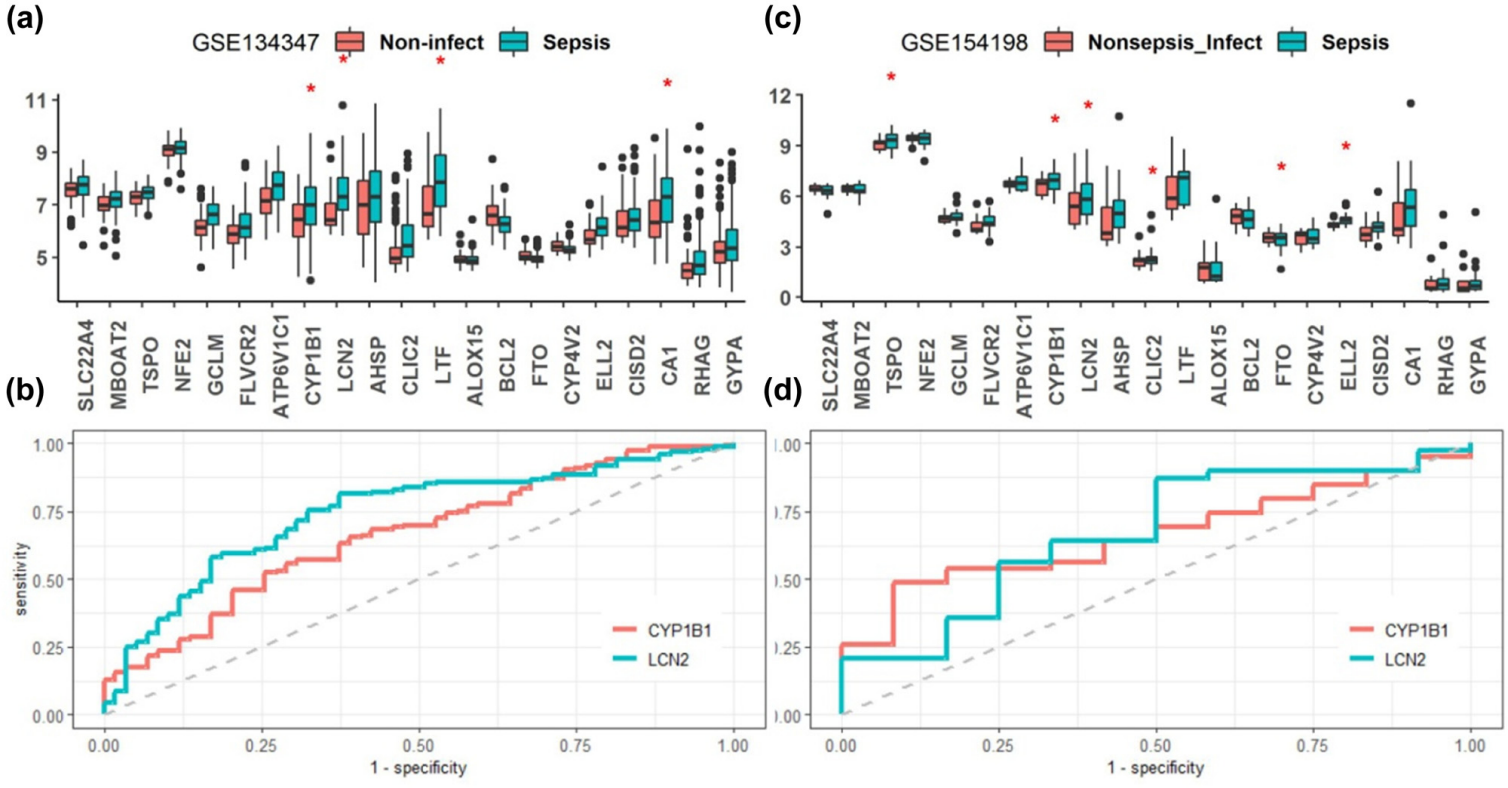
Expression levels of the sepsis-related iron metabolism-related genes in sepsis vs noninfectious disease (a), and in sepsis vs nonsepsis (uncomplicated) infectious disease (c). ROC curves of LCN2 and CYP1B1 which simultaneously differentially expressed in the two datasets of GSE134347 (b) and GSE154198 (d). The AUC of LCN2 and CYP1B1 were 0.744 (0.671–0.816) and 0.666 (0.586–0.746) in GSE134347 dataset, and 0.671 (0.489–0.853) and 0.662 (0.506–0.819) in GSE154198 dataset (Table S2). *p < 0.05.

Next to further evaluate the diagnostic values of the iron metabolism-related genes in non-sepsis diseases, we first compared their expressions between sepsis and uncomplicated infections in GSE154198 datasets (Figure 7a). The genes TPSO, CYP1B1, LCN2, CLIC2, and ELL2 were up-regulated and FTO was down-regulated in sepsis compared to the uncomplicated infections (*p* < 0.05). Then, we compared the expressions of the 21 genes between sepsis and noninfectious disease in GSE134347 datasets (Figure 7c), and found that the genes CYP1B1, LCN2, LTF, and CA1 were up-regulated in sepsis than the noninfectious disease (*p* < 0.05). These results from the two datasets indicated that LCN2 and CYP1B1 expressions simultaneously distinguished sepsis from healthy controls, and also from uncomplicated infectious and noninfectious diseases. Further ROC analysis suggested the AUC of LCN2 and CYP1B1 in diagnosis between sepsis and uncomplicated infectious or noninfectious diseases were both >0.66 (Figure 7b and d). All the AUC values of the ROC in terms of sepsis vs non-sepsis controls are listed in Table S2.

### 3.4 Genetic signatures distinguished severe forms from mild forms of sepsis

**Figure 8:**
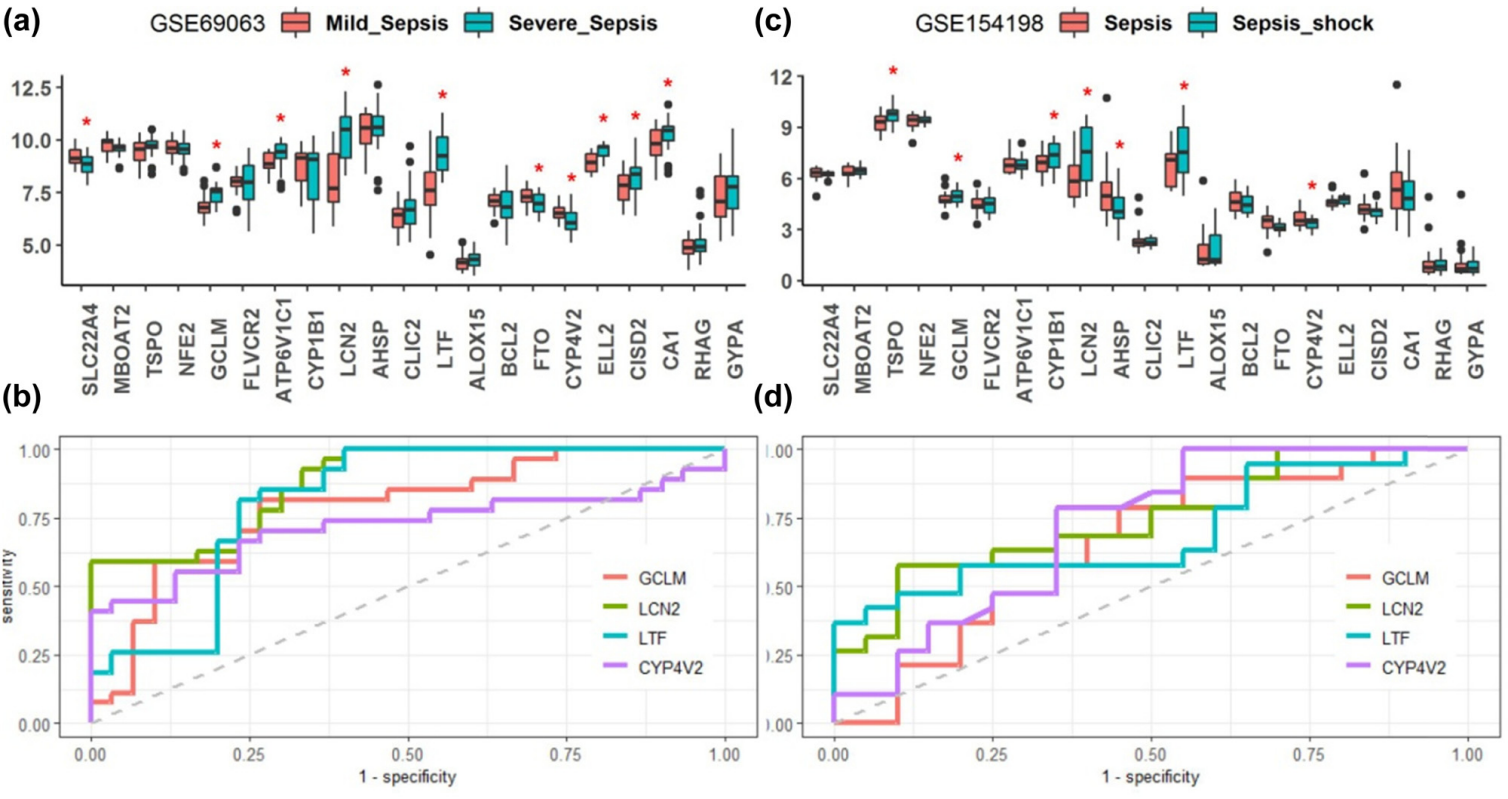
Expression levels of the sepsis-related iron metabolism-related genes in severe sepsis vs mild sepsis (a), and in sepsis shock vs sepsis (c). ROC curves of GCLM, LCN2, LTF, and CYP4V2 which simultaneously differentially expressed in GSE69063 dataset (b) and GSE154198 dataset (d). The AUC of GCLM, LCN2, LTF, and CYP4V2 were 0.788 (0.667–0.909), 0.88 (0.796–0.965), 0.815 (0.698–0.932), and 0.717 (0.572–0.863) in GSE69063 dataset, and 0.65 (0.471–0.829), 0.745 (0.588–0.901), 0.697 (0.526–0.869), and 0.721 (0.556–0.886) in GSE154198 dataset (Table S3). *p < 0.05.

To explore the diagnostic values of the genetic signatures between the severe forms and mild forms of sepsis, we compared the mRNA levels of the 21 iron metabolism-related genes between severe and mild sepsis in GSE69063 datasets, and between sepsis and sepsis shock in GSE154198 datasets, respectively (Figure 8a and c). When compared to the mild cases of sepsis, we found the SLC22A4, GCLM, ATP6V1C1, LCN2, LTF, ELL2, CISD2, and CA1 were up-regulated and the FTO and CYP4V2 were down-regulated in the severe cases of sepsis (*p* < 0.05). Additionally, when compared to sepsis shock, we found TPSO, GCLM, CYP1B1, LCN2, and LTF were up-regulated and the FTO and CYP4V2 were down-regulated in the sepsis (*p* < 0.05). These results from the two datasets indicated that GCLM, LCN2, LTF, and CYP4V2 expressions could consistently distinguish severe forms of sepsis from mild forms of sepsis. Further ROC analysis suggested that the AUC of GCLM, LCN2, LTF, and CYP4V2 in diagnosis between severe forms and mild forms of sepsis were all >0.65 (Figure 8b and d). All the AUC values of the ROC in terms of severe forms vs mild forms of sepsis are listed in Table S3.

**Figure 9:**
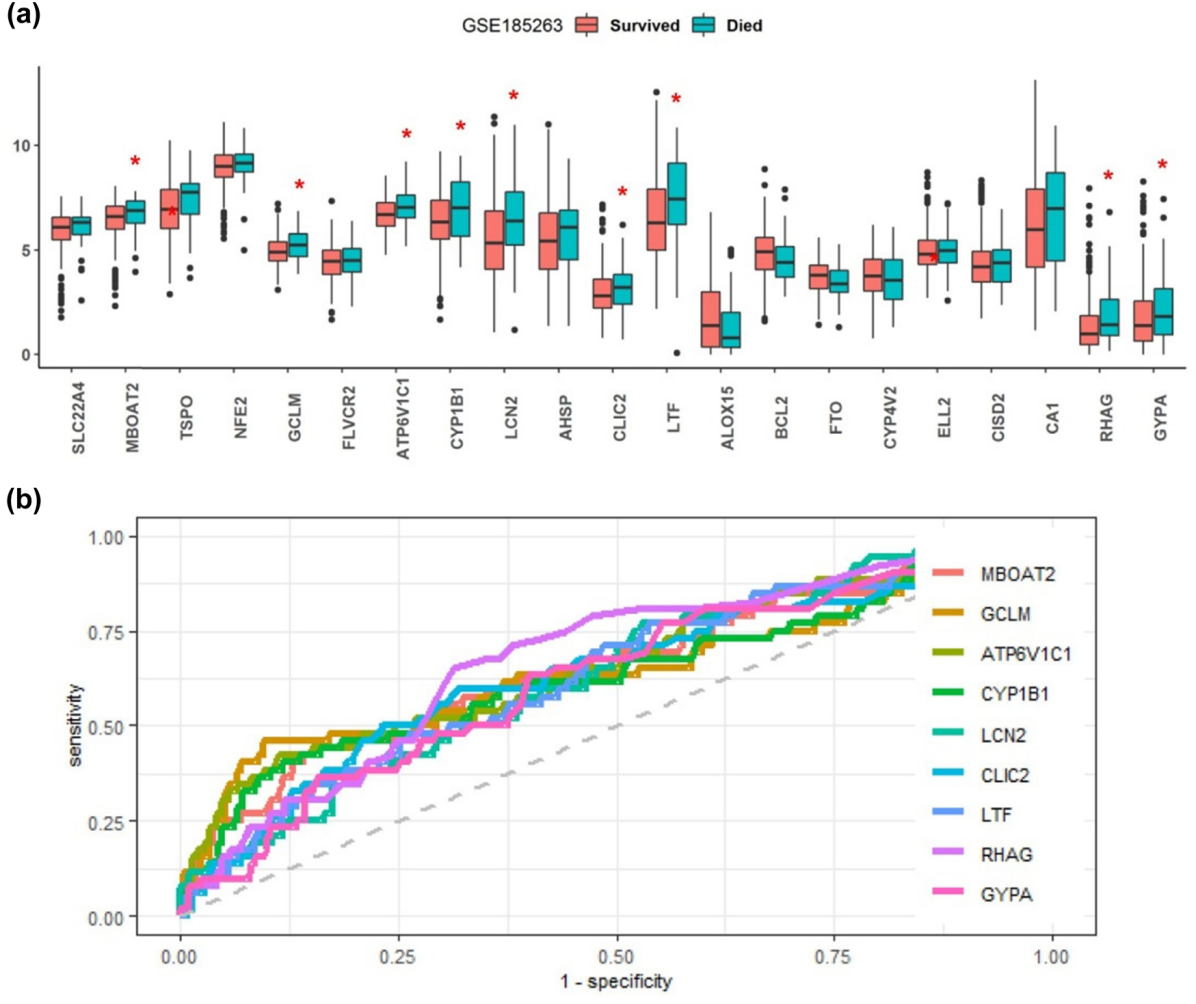
Expression levels of the sepsis-related iron metabolism-related genes between the different outcomes (a). ROC curves of MBOAT2, GCLM, ATP6V1C1, CYP1B1, LCN2, CLIC2, LTF, RHAG, and GYPA, which were up-regulated in the deceased patients than the survived cases in the GSE263298 dataset (b). The AUC values were MBOAT2: 0.656 (0.569–0.744), GCLM: 0.65 (0.555–0.745), ATP6V1C1: 0.664 (0.575–0.752), CYP1B1: 0.635 (0.543–0.727), LCN2: 0.631 (0.55–0.712), CLIC2: 0.637 (0.548–0.726), LTF: 0.632 (0.548–0.717), RHAG: 0.68 (0.602–0.758), GYPA: 0.626 (0.543–0.71) (Table S3). *p < 0.05.

Moreover, we also evaluated the predictive values of these 21 differentially expressed iron metabolism-related genes for fatal outcome in sepsis. When compared the gene expressions between the different outcomes in the GSE263298 dataset (Figure 9a), we found that MBOAT2, GCLM, ATP6V1C1, CYP1B1, LCN2, CLIC2, LTF, RHAG, and GYPA were all significantly up-regulated in the deceased patients than the survived cases. Further ROC analysis showed that the AUC of these nine genes in distinguishing the fatal outcome from the survived outcome were >0.62 (Figure 9b). All the AUC values of the ROC in terms of deceased vs survived sepsis are listed in Table S3.

## 4 Discussion

In sepsis, iron metabolism is altered in the host as a conserved strategy to combat invading pathogens [6]. Although iron retention has protective effects, an increase in labile iron in the host may also cause oxidative injury and cell death (ferroptosis etc.), and the iron disorders are substantial and correlate with the severity of sepsis, which suggests that iron and iron metabolism-related genes may be useful as diagnostic markers for evaluating the severity and predicting the outcome of the disease [6]. In the present study, by combining the four blood transcriptome datasets in GEO database, we identified 21 sepsis associated iron metabolism-related genes, including 9 transporters, 8 enzymes, and 4 regulatory factors, among which 17 were found to be up-regulated and 4 were down-regulated in sepsis.

Among these differentially expressed iron metabolism-related genes, LCN2 is the only gene that showed significant difference simultaneously in all the comparisons including sepsis vs healthy controls, sepsis vs non-infection disease, sepsis vs uncomplicated infections, sepsis shock vs sepsis, severe sepsis vs mild sepsis, and also in the comparisons of deceased vs survived group of sepsis, suggesting its expression has a high diagnostic value for sepsis and also for the severe forms of sepsis like sepsis shock. LCN2 is an innate immune protein, and is an iron-trafficking protein. It has emerged as a critical iron regulatory protein during physiological and inflammatory conditions [15]. As a bacteriostatic factor, LCN2 obstructs the siderophore iron-acquiring strategy of bacteria and thus inhibits bacterial growth. In addition, as part of host nutritional immunity, LCN2 facilitates systemic, cellular, and mucosal hypoferremia during inflammation. LCN2 deficiency dysregulates the host iron homeostasis and exacerbates endotoxin-induced sepsis [16], therefore development of a therapeutic strategy targeting lipocalin-2 could be highly promising in the management of gut-origin sepsis [17]. Plasma LCN2 also has a high sensitivity and a high negative predictive value for detection of AKI in adult sepsis patients [18].

Similar with LCN2, LTF is also an iron-binding and multifunctional protein among the sepsis associated iron metabolism-related genes identified in this study. LTF is secreted by exocrine glands and by neutrophils, and is a key element of host defenses [19]. Our analysis suggested that LTF was differentially expressed in the comparisons of sepsis vs healthy controls, sepsis vs non-infection disease, sepsis shock vs sepsis, severe sepsis vs mild sepsis, and also in the comparisons of deceased vs survived group of sepsis. LTF is emerging as a potent regulator of iron and inflammatory homeostasis, which is the sole glycoprotein able to contemporarily act against microbial multiplication, biofilm formation, iron disorders, oxidative stress, and viral and parasitic infections as well as inflammation [20]. Besides, FLVCR is also an iron/heme binding protein, which plays a central role for free heme in the pathogenesis of severe sepsis [8]. Our results suggested that FLVCR was only differentially expressed in the comparisons of sepsis vs healthy controls. As a heme exporter, FLVCR regulates heme synthesis and degradation and controls activity of cytochromes P450 [21]. In heme-Hb metabolic pathways, we also identified AHSP, which is a small protein that binds specifically to the α Hb subunit, as an up-regulated gene in sepsis. AHSP mainly functions to regulate the stability, folding, and assembly of the α Hb subunit [22].

In our results, two cytochromes were identified as DEGs in sepsis P450, CYP1B1, and CYP4V2. CYP1B1 was found to be up-regulated simultaneously in sepsis vs healthy controls, sepsis vs non-infection disease, and sepsis vs uncomplicated infections, while CYP4V2 was down-regulated simultaneously in sepsis vs healthy controls, sepsis shock vs sepsis, and severe sepsis vs mild sepsis. These two genes show opposite changes in sepsis. CYP1B1 is a heme-thiolate monooxygenase, which is involved in NADPH-dependent phase I metabolism of a variety of xenobiotics [23]. CYP4V2 is a microsomal fatty acid omega-hydroxylases that function together with mitochondrial and peroxisomal beta-oxidation enzymes to degrade cellular lipids [24]. The detailed mechanism for the two genes in sepsis still need to be clarified.

Except for CYP4V2, there were also three genes down-regulated in sepsis, including ALOX15, BCL2, and FTO. ALOX15 (or 12/15-LOX) is an enzyme, which oxidizes mainly omega-6 and omega-3 fatty acids, to generate a number of bioactive lipid metabolites. ALOX15 has been reported to play an important role in oxidative and inflammatory responses, as its metabolite 12(S)-HETE is a potent, pro-inflammatory chemoattractant for neutrophils and leukocytes [25]. BCL2 is a key player in multiple cellular processes, chief amongst them being an inhibitor of autophagy, suppresses apoptosis by controlling the mitochondrial membrane permeability [26]. FTO acts as oxidative demethylases of N6-methyladenosine (m6A). In sepsis, the down-regulation of FTO leads to evidently increasing the total levels of m6A modification upon exposure to ferroptosis-inducing compounds [27].

Ferroptosis has been widely reported to be involved in sepsis occurrence and development [28]. Carbonic anhydrases play a crucial role in ferroptosis, for their regulations on iron solubility and iron transporter activities, and functioning in lipogenesis with protective effects on lipid peroxidation [29]. CA1 (carbonic anhydrase 1) was found to be up-regulated in sepsis from our results. In addition, upon ferroptosis, GCLM, the first rate limiting enzyme of glutathione (GSH) synthesis, increases in response to oxidative stress conditions [30]. Our results revealed that GCLM was significantly up-regulated simultaneously in the comparisons of sepsis vs healthy controls, sepsis shock vs sepsis, severe sepsis vs mild sepsis. This up-regulation was thought to be mediated through transcriptional factors, such as Nrf2 [31].

Interestingly, two transcriptional factors were identified as up-regulated genes in sepsis from our analysis: NFE2 and ELL2. NFE2 plays a pivotal role in the transcription control of erythroid-specific genes, including β-globin, and is necessary for the transcriptional activation of heme biosynthetic enzymes PBGD3 and FECH [32]. NFE2 is a novel regulator of pro-oxidant induced oxidative stress [33]. ELL2 is an elongation factor component of the super elongation complex (SEC), involved in snRNA transcription. ELL2 directs immunoglobulin secretion in plasma cells by stimulating altered RNA processing [34].

In addition to the above genetic signatures, we also identified several membrane transporters, which were all up-regulated in sepsis, including SLC22A4, TPSO, and ATP6V1C1. Among them, SLC22A4 (or OCTN1) belongs to a small sub-family of organic cation transporters. It is expressed in several districts including peritoneum, and involved in control of inflammatory processes that are critical in this district [35]. TSPO is an outer mitochondrial membrane protein that is widely used as a biomarker of neuroinflammation [36]. In sepsis, cognitive damage and microglial activation could be reduced by modulation of TSPO by antagonist PK-11195 [37]. ATP6V1C1, which is V-ATPase subunit, are essential ATP-dependent proton pumps. V-ATPase is generally thought to play a terminal, degradative role in autophagy-related processes [38].

From our analysis, there are also several other iron metabolism-related genes up-regulated in sepsis. CLIC2, as the least investigated CLIC family member, is expressed in normal blood vessel endothelial cells, while involving in the maintenance of the vascular barrier functions [39]. And CISD2 (NAF-1), which plays a key role in regulating cellular homeostasis, is reported to control calcium, reactive oxygen species (ROS), and iron signaling mechanisms [40]. And MBOAT2 is an acyltransferase and previously found to be associated with multiple diseases like cardiomyopathy, multiple sclerosis, adrenomyeloneuropathy, and pancreatic Cancer [41]. These genes conceivably play some important roles in sepsis, but the specific mechanisms need to be further studied.

In conclusion, we identified the genetic signatures related to the iron-metabolism in sepsis by using a bioinformatics analysis of the blood transcriptomic datasets. A total of 21 iron metabolism-related signatures were identified including 9 transporters, 8 enzymes, and 4 regulatory factors, among which LCN2 showed consistent differences between sepsis and healthy groups, sepsis and non-sepsis groups, and mild and severe forms of sepsis. These iron metabolism-related genetic signatures may represent diagnostic and therapeutic biomarkers of sepsis, and the knowledge of these genes will improve our understanding of the molecular mechanism underlying the occurrence of sepsis.

## Supplementary Material

Supplementary Figures

Supplementary Tables
