# Supplementary figures and images for: Identification of iron metabolism-related genes as diagnostic signatures in sepsis by blood transcriptomic analysis
Source: Open Life Sci. 2023 Feb 9;18(1):20220549. doi: 10.1515/biol-2022-0549 (PMC9938542; doi:10.1515/biol-2022-0549)

Figure S1 - GSE69063

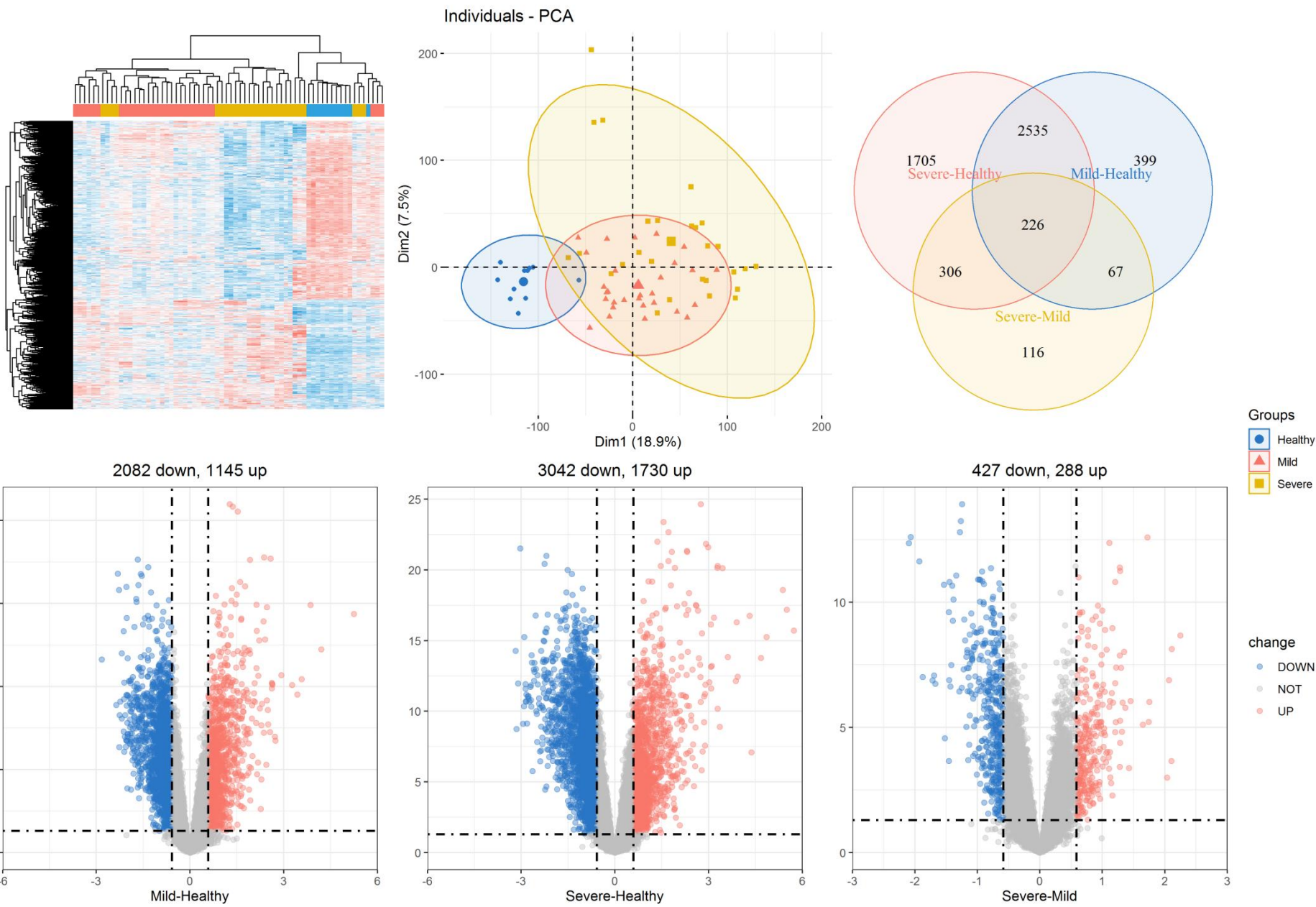

Figure S2 - GSE154198

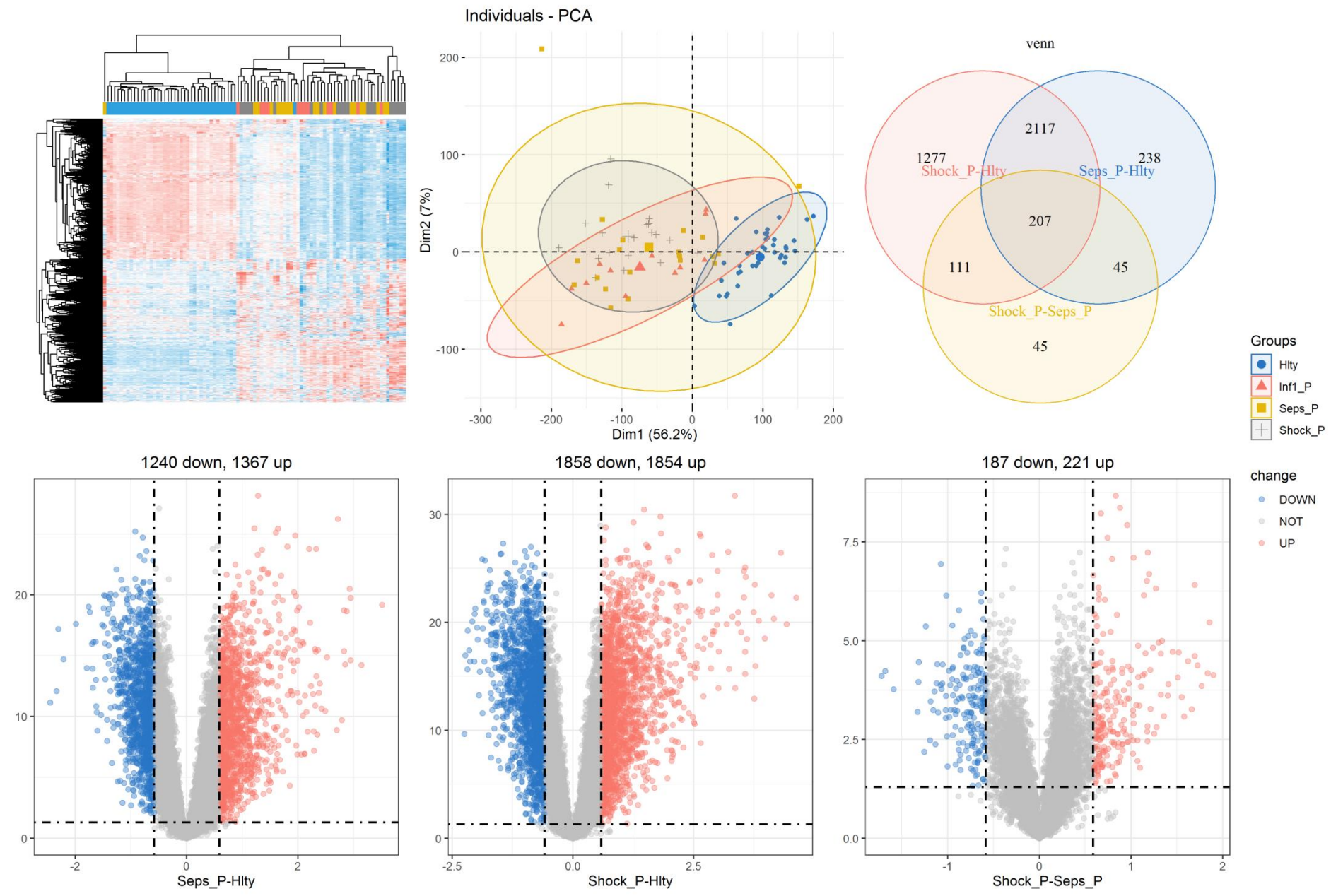

Figure S3 - GSE134347

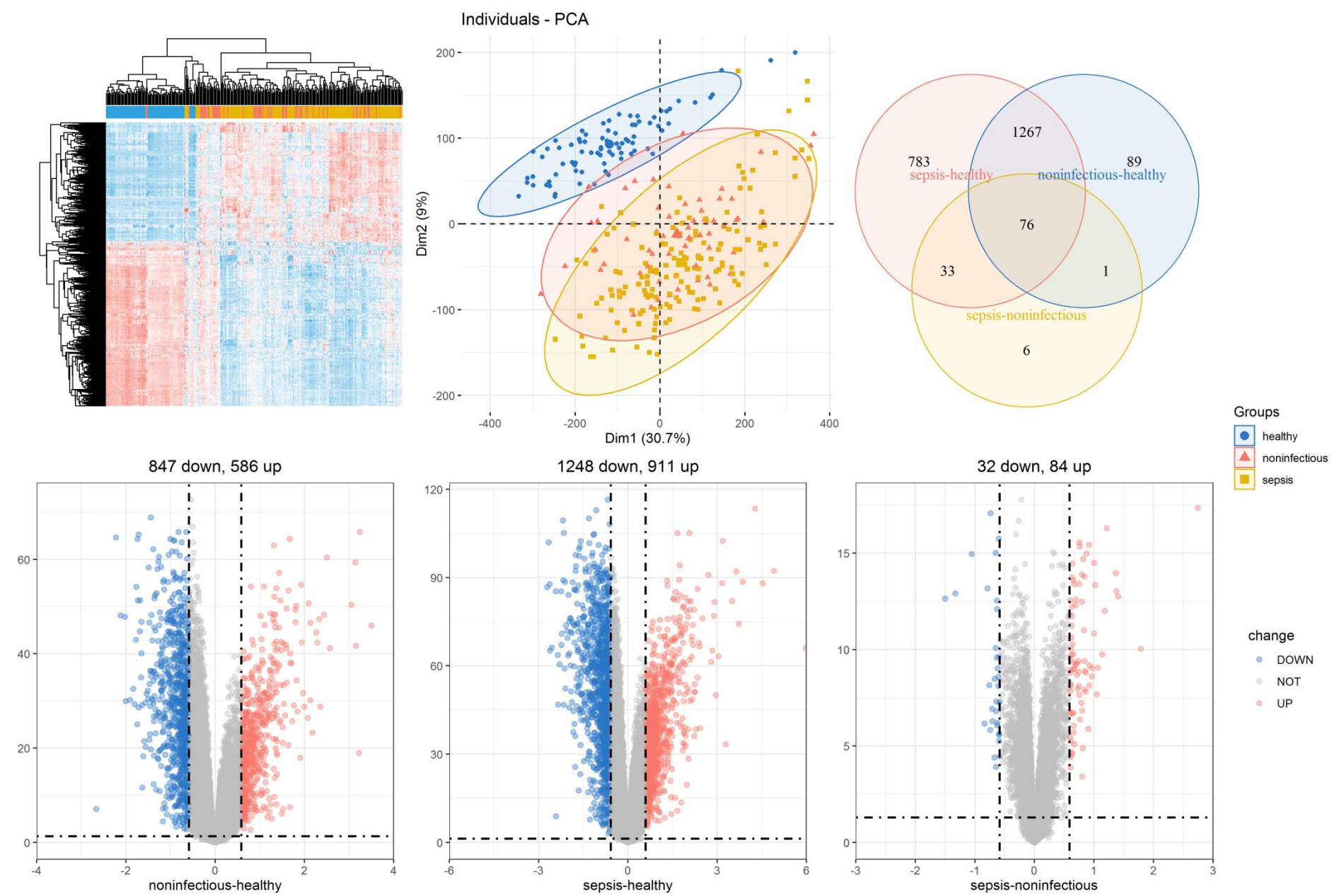

Figure S4 - GSE185263

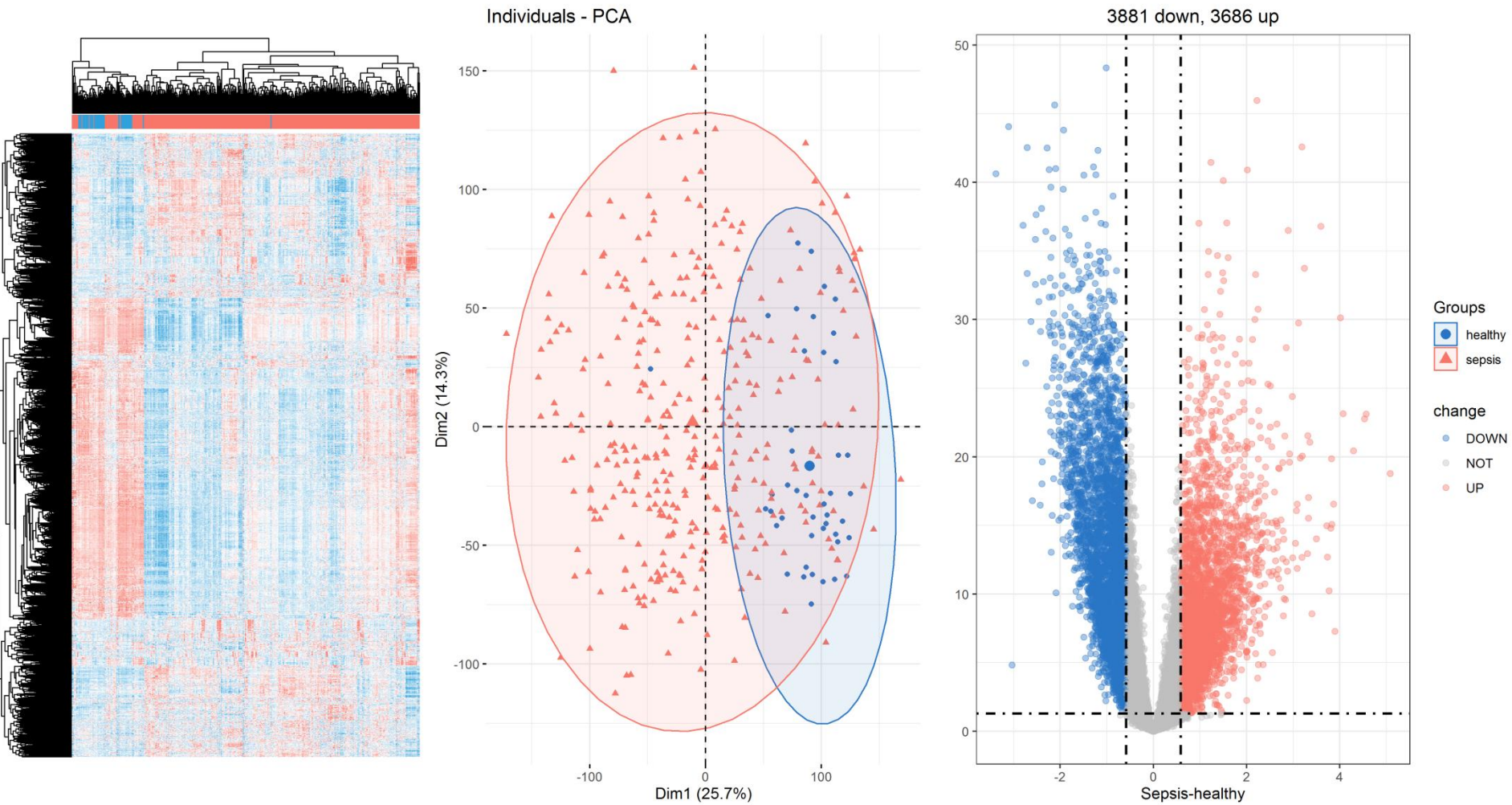

Supplement: Supplementary Figures [file biol-2022-0549-sf.pdf]
